# Obesity in children & adolescents

**Published:** 2010-11

**Authors:** Manu Raj, R. Krishna Kumar

**Affiliations:** *Department of Pediatric Cardiology Amrita Institute of Medical Sciences & Research Centre, Kochi, Kerala, India*

**Keywords:** Adolescents, children, dietary management, obesity, overweight

## Abstract

Worldwide, obesity trends are causing serious public health concern and in many countries threatening the viability of basic health care delivery. It is an independent risk factor for cardiovascular diseases and significantly increases the risk of morbidity and mortality. The last two decades have witnessed an increase in health care costs due to obesity and related issues among children and adolescents. Childhood obesity is a global phenomenon affecting all socio-economic groups, irrespective of age, sex or ethnicity. Aetiopathogenesis of childhood obesity is multi-factorial and includes genetic, neuroendocrine, metabolic, psychological, environmental and socio-cultural factors. Many co-morbid conditions like metabolic, cardiovascular, psychological, orthopaedic, neurological, hepatic, pulmonary and renal disorders are seen in association with childhood obesity. The treatment of overweight and obesity in children and adolescents requires a multidisciplinary, multi-phase approach, which includes dietary management, physical activity enhancement, restriction of sedentary behaviour, pharmacotherapy and bariatric surgery. A holistic approach to tackle the childhood obesity epidemic needs a collection of activities including influencing policy makers and legislation, mobilizing communities, restructuring organizational practices, establishing coalitions and networks, empowering providers, imparting community education as well as enriching and reinforcing individual awareness and skills. The implications of this global phenomenon on future generations will be serious unless appropriate action is taken.

## Introduction

Worldwide, disease profiles are transforming at a rapid pace catching the attention of medical professionals and policy makers alike. This is particularly true in low and middle-income countries that form the major chunk of global population. The emerging epidemics of obesity, cardiovascular disease (CVD) and diabetes form the crux of this phenomenal change. Among these entities, obesity has become a colossal epidemic causing serious public health concern and contributes to 2.6 million deaths worldwide every year^1^. Obesity is an independent risk factor for CVD. Obesity is associated with an increased risk of morbidity and mortality as well as reduced life expectancy. The last two decades of the previous century have witnessed dramatic increase in health care costs due to obesity and related issues among children and adolescents^2^.

For children and adolescents, overweight and obesity are defined using age and sex specific normograms for body mass index (BMI). Children with BMI equal to or exceeding the age-gender-specific 95^th^ percentile are defined obese. Those with BMI equal to or exceeding the 85^th^ but are below 95^th^ percentiles are defined overweight and are at risk for obesity related co-morbidities^3^.

## Epidemiology

Childhood obesity affects both developed and developing countries of all socio-economic groups, irrespective of age, sex or ethnicity. It has been estimated that worldwide over 22 million children under the age of 5 are obese, and one in 10 children is overweight^4^. A wide range of prevalence levels exist, with the prevalence of overweight in Africa and Asia averaging well below 10 per cent and in the Americas and Europe above 20 per cent. The proportion of school-age children affected will almost double by 2010 compared with the most recently available surveys from the late 1990s up to 2003^4^. Obesity has become a serious public health concern affecting a significant portion of the population in countries like the US. Overall, among adults aged at least 20 yr in 1999-2002, 65.1 per cent were overweight and 30.4 per cent were obese. Among children aged 6 through 19 yr in 1999-2002, 31.0 per cent were overweight and 16.0 per cent were obese^5^. Asian countries are not immune to this phenomenon. For example, in China, the prevalence of overweight and obesity among children aged 7-9 yr increased from 1-2 per cent in 1985 to 17 per cent among girls and 25 per cent among boys in 2000^6^. In addition, obesity prevalence varies across socio-economic strata. In developed countries, children of low socio-economic status are more affected than their affluent counterparts^7^. The opposite is observed in developing countries: children of the upper socio-economic strata are more likely than poor children to be obese^8^,^9^. Indian data regarding current trends in childhood obesity are emerging. A recent study conducted among 24,000 school children in south India showed that the proportion of overweight children increased from 4.94 per cent of the total students in 2003 to 6.57 per cent in 2005 demonstrating the time trend of this rapidly growing epidemic^10^. Socio-economic trends in childhood obesity in India are also emerging. A study from northern India reported a childhood obesity prevalence of 5.59 per cent in the higher socio-economic strata when compared to 0.42 per cent in the lower socio-economic strata^11^.

## Aetiopathogenesis of childhood obesity

Aetiopathogenesis of childhood obesity is multi- factorial. Interactions between genetic, neuroendocrine, metabolic, psychological, environmental and socio- cultural factors are clearly evident in childhood obesity.

### Gene mutations and obesity

Single and polygenic gene mutations that occur naturally can produce obesity in rodents like mice and rats. The prototypic obese mice with single gene defects are the obese (ob/ob, Lep^ob^) and diabetes (db/db, Lepr^db^) autosomal recessive mutations. These mutations produce phenotypes of severe hyperphagia, obesity, type 2 diabetes, defective thermogenesis, and infertility. The mutant gene responsible for the phenotype in Lep^ob^mice encodes a protein termed leptin, which is deficient in these animals^3^. Leptin deficiency has been documented in subsets of human obesity^3^. Severe early-onset human obesity caused by a mutant leptin receptor has also been identified^3^. In the fatty (fat/fat) mouse, the recessively inherited mutation causes hyperinsulinaemia without hyperglycaemia and post-pubertal obesity that is less severe than that seen in ob/ob or db/db mice. The yellow mutation of agouti mice is a dominant trait that causes yellow coat colour, obesity, and diabetes^3^. The polygenic mouse models of obesity closely resemble the human obesity phenotypes than single gene models and have mutations that influence obesity, plasma cholesterol levels, body fat distribution, and propensity toward development of obesity on a high-fat diet^3^.

Genetic conditions known to be associated with predilection for obesity include Prader-Willi syndrome, Bardet-Biedl syndrome, and Cohen syndrome. Obesity clearly demonstrates a familial tendency. The Avon Longitudinal Study demonstrated that the odds of children aged 7 becoming obese if the father, mother or both had obesity were 2.93, 4.66 and 11.75, respectively showing clearly the dominant influence of parental obesity^12^. Before 3 yr of age, parental obesity is a stronger predictor of obesity in adulthood than the child’s weight status^13^.

### Neuroendocrinology of energy metabolism

Energy metabolism is controlled by complex neuroendocrine interactions, which influence food intake and energy expenditure. Leptin, almost exclusively produced by the adipose tissue is the major hormone in this mechanism that acts centrally in the hypothalamus. Low plasma concentrations of leptin and insulin as found during fasting and weight loss increase food intake and decrease energy expenditure by stimulating neuropeptide Y synthesis, and perhaps by inhibiting sympathetic activity and other catabolic pathways^3^. High leptin and insulin concentrations found during feeding and weight gain decrease food intake and increase energy expenditure through release of melanocortin and corticotropin-releasing hormone, among others. The major peptides that stimulate feeding are orexins A and B, which are secreted by the hypothalamus, and ghrelin, which is secreted by the stomach^3^.

### Fundamental phases in evolution of obesity

There are critical phases in the evolution of obesity. Intrauterine growth patterns play a significant role in the evolution of obesity by modifying fat and lean body mass, neuroendocrine appetite control mechanisms, and pancreatic functional capacities. Longitudinal studies have identified a strong relationship between birth weight and BMI attained in later life. Increasing birth weight was independently and linearly associated with increasing prevalence of childhood obesity in the Avon Study^12^. In addition, low birth weight babies show a dramatic transition to central adiposity and insulin resistance very early in life^14^. These two factors are known to increase cardiovascular risk manifold^14^. Catch up growth and early adiposity rebound increase the odds of children as well as adults becoming obese significantly^12^,^15^. The combination of lower birth weight and higher attained BMI is most dangerous as it is associated with extreme CVD risk in later life^16^.

The nature and duration of breastfeeding have been found to be negatively associated with risk of obesity in later childhood^17^,^18^. A systematic review of nine studies has concluded that breastfeeding seems to have a small but consistent protective effect against obesity in children^19^. The normal pattern of insulin resistance during early puberty may be a natural cofactor for unnecessary weight gain as well as various co-morbidities of obesity^20^. Early menarche is clearly associated with extent of obesity, with a two-fold increase in rate of early menarche associated with BMI greater than the 85^th^ percentile^21^. The risk of obesity persisting into adulthood is higher among obese adolescents than among younger children^13^. Observations suggest that up to 80 per cent of overweight adolescents will become obese adults^22^.

### Environmental risk factors for obesity

Environmental risk factors for overweight and obesity are very strong and inter-related. Sub-optimal cognitive stimulation at home and poor socio-economic status predict development of obesity^23^. Parental food choices significantly modify child food preferences^24^, and degree of parental adiposity is a surrogate for children’s fat preferences^25^. Children and adolescents of poor socio-economic status tend to consume less quantities of fruits and vegetables and to have a higher intake of total and saturated fat^26^–^28^. Early rebound of BMI is linked to glucose intolerance and diabetes in adults^29^. Short sleep duration in children is also associated with an increase in the odds of becoming obese as well as an increase in body fat per cent^30^.

### Societal changes and obesity

Dramatic and rapid societal changes during the last decades have contributed significantly to childhood obesity. There is evidence stating that individual’s eating and physical activity behaviours are heavily influenced by surrounding social and physical environmental contexts both for adults and children. Urbanization related intake behaviours that have been shown to promote obesity include frequent consumption of meals at fast-food outlets^31^,^32^, consumption of oversized portions at home and at restaurants^33^,^34^, consumption of high calorie foods, such as high-fat, low-fiber foods^35^,^36^, and intake of sweetened beverages^37^,^38^. These behaviours are cultivated in an environment in which high calorie food is abundant, affordable, available, and easy to consume with minimal preparation as is the case of urban cities throughout the country. Television viewing and other sedentary activities have also been related to childhood obesity^39^,^40^. Unfortunately this habit is growing exponentially in developing countries as well. Low levels of physical activity is definitely promoted by an automated and automobile-oriented environment that is conducive to a sedentary lifestyle^41^. Community design and infrastructure characteristics are also becoming increasingly important in determining levels of obesity in populations^42^. Such factors include availability of safe walkways, bicycle paths, playgrounds and other avenues for physical activity related recreation.

## Co-morbidities related to obesity

Obesity is associated with a number of co-morbidities in adolescents and children. Some common co-morbid conditions related to obesity in adolescents and children are presented in the Table.

**Table:** Adverse outcomes in childhood obesity

Cardiovascular	High blood pressure
	Early onset of atherosclerosis
	Left ventricular hypertrophy
Endocrine	Insulin resistance
	Diabetes mellitus (NIDDM)
	Menstrual abnormalities
	Polycystic ovarian syndrome (PCOS)
Gastrointestinal	Gallstones
	Non alcoholic steatohepatitis (NASH)
	Hepatic fibrosis
	Cirrhosis
Neurological	Pseudotumor cerebri
Orthopedic	Slipped capital femoral epiphysis
	Tibia Vara
	Osteoarthritis
Psychosocial	Obsessive concern about body image
	Expectation of rejection
	Progressive withdrawal
	Low self esteem
	Depression
Pulmonary	Increased bronchial hyperactivity
	Asthma exacerbation
	Obstructive sleep apnoea
	Pickwickian syndrome
	Pulmonary embolism
Renal	Increased sensitivity to sodium
	Decreased natriuresis
	Proteinuria
	Focal segmental glomerulosclerosis (FSGS)

### Metabolic syndrome

Metabolic syndrome is defined as a constellation of risk factors, including obesity, dyslipidaemia, impaired glucose metabolism and elevated blood pressure, all major predictors for cardiovascular disease^43^. It has been proven by previous studies that cardio metabolic risk factors frequently cluster in obese children and adolescents. Goodman *et al*^44^ identified four clusters of risk factors in adolescents and found that obesity had the most substantial influence on cumulative cardio metabolic risk. Each component of the syndrome worsens with increasing obesity independent of age, sex, and pubertal status^45^.

The trigger factor for initiation of events leading to metabolic syndrome in obesity is not clearly identified. Two schools of thought predominate, one focusing on intra-abdominal fat depots and the other on insulin resistance as starting points. Accumulation of visceral fat is characterized by high lipid turnover resulting in higher levels of free fatty acids (FFA) in the portal circulation^46^. This could lead to enhanced lipid synthesis, gluconeogenesis, insulin resistance and activation of sympathetic nervous system^47^–^50^. Activation of sympathetic nervous system can contribute to elevation of blood pressure through its effects on vascular tissue as well as renal handling of sodium and water^51^,^52^. Insulin resistance can independently lead to increased hepatic synthesis of very low-density lipoprotein (VLDL), resistance of the action of insulin on lipoprotein lipase in peripheral tissues, enhanced cholesterol synthesis, increased high-density lipoprotein (HDL) degradation, increased sympathetic activity, proliferation of vascular smooth muscle cells, and increased formation and decreased reduction of plaque^22^. The prevalence of metabolic syndrome in obese children and adolescents vary with the type of diagnostic definition used as well as the population studied. Evidence from large international studies suggests that it could range from 10 to 40 per cent depending on the levels of obesity^53^. Similar trends were reported from adolescent Indian population as well^54^.

### Type 2 diabetes mellitus

The association of obesity with type 2 diabetes in adolescents and children is very strong and confirmed by various studies. Evidence entail that obesity driven type 2 diabetes might become the most common form of newly diagnosed diabetes in adolescent youth within 10 years^55^. Evidence is accumulating which suggests a global spread of type 2 diabetes in childhood^56^. Traditionally type 2 diabetes mellitus had been a disease of adults; however, the same now occurs in increased numbers among obese adolescents^22^. Studies demonstrate an increased risk of nephropathy and retinopathy compared to young people with type 1 diabetes, while recent data indicate early signs of cardiovascular disease in youth with type 2 diabetes^57^–^59^. Evidence is emerging of a growing prevalence of type 2 diabetes among urban Indian children as well^60^.

### Cardiovascular abnormalities

Obesity significantly contributes to morbidity and mortality from cardiovascular disease. Obesity may affect the heart through its influence on known risk factors such as dyslipidaemia, hypertension, glucose intolerance, inflammatory markers, obstructive sleep apnoea/hypoventilation, and the prothrombotic state, as well as through yet unrecognized mechanisms. Landmark studies like Bogalusa, Muscatine and Cardiovascular risk in young Finns study have demonstrated that obesity during childhood and adolescence is a determinant of a number of cardiovascular risk factors in adulthood^61^–^63^. Studies have demonstrated significant association of obesity with hypertension in children and adolescents^10^,^64^. These studies have shown that the association is stronger in case of systolic hypertension than that of diastolic hypertension. Left ventricular hypertrophy, a well-known cardiovascular risk factor has an association with obesity even from childhood which tracks and becomes stronger in young adulthood^65^. Emerging cardiovascular risk factors like carotid intima media thickness as well as carotid elasticity has also shown strong association with childhood obesity^63^. Obstructive sleep apnoea, a well-known cardiovascular risk factor is also associated with obesity in children and has also shown to induce insulin resistance. Treatment of this condition improves lipid profiles, C-reactive protein, and apolipoprotein B which confirms its pathogenic role in lipid homeostasis and systemic inflammation^66^.

### Psychosocial abnormalities

Psychosocial abnormalities are closely associated with obesity in children and adolescents. Obesity in adolescence may be associated with later depression in adulthood^67^. In addition, abdominal obesity seems to be strongly associated with concomitant depression in males. Though both sexes can be affected by obesity-induced depression, females demonstrate a more robust association. Females obese as adolescents may be at increased risk for development of depression or anxiety disorders^68^. Among obese children, appearance related teasing is more frequent and upsetting. Degree of teasing is associated with higher weight concerns, more loneliness, poor self-perception of physical appearance, higher preference for sedentary or isolated activities and lower preference for social activities^69^. Overeating among adolescents is associated with a variety of adverse behaviours and negative psychological experiences including low self-esteem and suicidal tendencies^70^. The association of suicidal tendencies is stronger in those meeting the criteria for binge eating syndrome.

## Treatment of obesity

The treatment of overweight and obesity in children and adolescents requires a multidisciplinary approach with a holistic outlook. The team should include a paediatric physician, nurse practitioner, dietician, physical instructor, behavioural therapist and a social worker in addition to a motivated team of parents, caretakers, teachers and policy makers. The immediate goal is to bring down the rate of weight gain, followed by a period of weight maintenance and finally weight reduction to improve BMI. The long-term goal is to improve quality of life and reduction in morbidity as well as mortality associated with overweight and obesity.

### Targets for obesity treatment

No targets are defined for treating children less than two years who have overweight or obesity. For overweight children in the age group of 2-5 yr weight maintenance is all that is required. For obese children in the same group, weight maintenance is attempted. A minimal weight loss of 0.5 kg/month may be permitted if it occurs with a balanced diet supplying adequate calories^71^. For overweight children in the age group of 6-11 yr weight maintenance is adequate. For obese children in the same group, weight maintenance or a minimal weight loss of 0.5 kg/month may be attempted. If the child’s BMI is more than 99^th^ percentile, a moderate weight loss of not more than 1 kg/wk may be attempted. For overweight adolescents in the age group of 12-18 yr weight maintenance is adequate. For obese adolescents in the same group, a moderate weight loss not more than 1 kg/wk may be attempted^71^.

### Components and phases of obesity treatment

The components of overweight and obesity treatment include dietary management, physical activity enhancement, restriction of sedentary behaviour, pharmacotherapy and bariatric surgery. The various phases of obesity management in ascending order of intensity include prevention oriented approach, structured weight management, comprehensive multidisciplinary intervention and tertiary care intervention. Each component goes through the various phases as required.

### Dietary management

Dietary management should aim at weight maintenance or weight loss without compromising appropriate calorie intake and normal nutrition. Due emphasis should be given to initiate and maintain healthy eating patterns. A standard protocol is to recommend a fat intake of 30 to 40 per cent kcal in children 1 to 3 yr old, with a reduction to 25 to 35 per cent in children 4 to 18 yr old; a carbohydrate intake of 45 to 65 per cent kcal in all children and adults; and protein intakes of 5 to 20 per cent kcal in children 1 to 3 yr old with gradual increase to 10 to 30 per cent kcal in children 4 to 18 yr old^72^.

In obese children 8 yr or older, the Dietary Intervention Study in Children (DISC) intervention diet can be introduced without compromising growth, development and pubertal maturity^73^. This diet distributes 58 per cent of total calorie intake to carbohydrates, 28 per cent to fats and 14 per cent to protein. Of the 28 per cent calories from fats, 11 per cent should be from monounsaturates, 9 per cent from polyunsaturates and less than 8 per cent from saturates. Cholesterol intake should be less than 75 mg/1000 kilocalories, not to exceed 150 mg per day. Age-appropriate serving sizes including 5 or more servings of fruit and vegetables, 3 or more servings of low fat milk or dairy products, and 6 or more servings of whole-grain and grain products per day as well as adequate amounts of dietary fiber (age in yr + 5 g/d) should also be encouraged^22^.

Due emphasis should be given to reduction of eat outs, planning for healthy snacks, balanced diet, adequate intake of fruits and vegetables, fiber content of diet and avoidance of high calorie/high fat foods. The benefits of salt reduction, restriction of sugar rich beverages and avoidance of trans fatty acids from the diet are supported with strong evidence^74^–^76^.

### Physical activity enhancement

Moderate intensity regular physical activity is essential for the prevention of overweight and obesity as well as for treatment of the same. Children and adolescents should engage in not less than 60 min of moderate to vigorous physical activity per day to achieve optimum cardiovascular health^77^. Overweight and obese children should target higher levels to achieve similar results. Longer periods of moderate intensity exercises like brisk walking burn more fat as calories and are excellent for reducing body fat^78^. Children should be prescribed physical activity that is safe, developmentally appropriate, interesting, practical and has a social element. Involving other members of the family in the exercise programme and supervising the activity on a regular basis will improve compliance. In addition to weight reduction, exercise training is associated with beneficial changes in fat and lean body mass, cardiovascular fitness, muscular strength, endothelial function and glucose metabolism, all of which significantly reduce the morbidity associated with excess weight^79^.

### Restriction of sedentary behavior

Children and adolescents typically indulge in sedentary activity like watching TV, sitting in front of computers and video games. Every hour of sedentary activity increases the chance of obesity and is also contributory to failure of many weight reduction attempts in adolescents and children. Screen time should be restricted to less than two hours per day as the opposite is associated with increased adiposity and higher weight status^80^. In addition, television viewing during early childhood predicts adult body mass index, which reinforces the long-term benefits of reducing screen time in young age^81^. Excessive TV viewing is associated with higher intakes of energy, fat, sweet and salty snacks and carbonated beverages in addition to reducing consumption of fruits and vegetables^82^. This makes TV time restriction an excellent opportunity to complement dietary management.

### Pharmacological treatment

Data supporting the use of pharmacological therapy for paediatric obesity are limited. The drugs sibutramine, orlistat and metformin are currently in use among obese children and adolescents with varying results. Sibutramine, a serotonin non adrenaline reuptake inhibitor enhances satiety and has been shown to be the most effective drug in treating adolescent obesity. This drug may be associated with side effects including increases in heart rate and blood pressure limiting its use in obese adolescents with higher blood pressure^83^,^84^. Orlistat, which is a pancreatic lipase inhibitor, acts by increasing faecal fat loss. It is associated with flatulence, diarrhoea, gallbladder diseases, malabsorptive stools and requires fat-soluble vitamin supplementation and monitoring^84^,^85^. Orlistat appears to be less effective in those who follow diets which are low in fats as is the case of many Indian diets. Metformin is a valuable adjuvant to the treatment of obese adolescents with severe insulin resistance, impaired glucose tolerance or polycystic ovarian syndrome^83^. Pharmacotherapy should be reserved as a second line of management and should be considered only when insulin resistance, impaired glucose tolerance, hepatic steatosis, dyslipidaemia or severe menstrual dysfunction persist inspite of lifestyle interventions.

### Surgical treatment

Many cases of severe adolescent obesity warrant aggressive approaches including surgical treatment. Adolescent candidates for bariatric surgery should be very severely obese (defined by body mass index of > 40), have attained a majority of skeletal maturity (generally > 13 yr of age for girls and > 15 yr of age for boys), and have co-morbidities related to obesity that might be remedied with durable weight loss^86^. More severe elevation of BMI (>50 kg/m^2^) may be an indication for surgical treatment in the presence of less severe co-morbidities. The bariatric procedures preferred in adolescents are Roux-en-Y gastric bypass and adjustable gastric banding. Late complications include small-bowel obstruction, incisional hernias, weight regain, as well as vitamin and micronutrient deficiencies. These patients warrant meticulous, lifelong medical supervision. Current evidence suggests that after bariatric surgery, adolescents lose significant weight and co-morbidities are appreciably reduced. Bariatric surgery performed in the adolescent period may be more effective treatment for childhood-onset extreme obesity than delaying surgery till adulthood^87^.

## Prevention of obesity

The ideal preventive strategy for obesity is to prevent children with a normal, desirable BMI from becoming overweight or obese. Preventive strategies should start as early as newborn period. The strategies may be attempted at the individual, community or physician’s level. Those at the individual level backed by consistent evidence include limiting sugar sweetened beverages, reducing daily screen time to less than two hours, removing television and computers from primary sleeping areas, eating breakfast regularly, limiting eating out especially at fast food outlets, encouraging family meals and limiting portion sizes^88^. Encouraging diets with recommended quantities of fruits and vegetables have been supported by mixed evidence. Healthy behaviours derived from this evidence include consuming a balanced diet rich in calcium and fiber, initiating and maintaining breastfeeding, accumulating 60 min or more of moderate to vigorous physical activity per day and limiting consumption of energy dense foods^71^.

Community level interventions include advocacy to increase physical activity at schools and at home through the creation of environments that support physical activity. These efforts could include creation and maintenance of parks, inclusion of child friendly walking and bicycle paths as well as creating awareness about locally available physical activity options. At the physician’s level it is essential to engage families with parental obesity or diabetes, because these children are at increased risk for developing obesity later in life^12^,^89^. It is also essential to encourage an authoritarian parenting style and to discourage a restrictive one. Physicians should encourage parents to be role models when it comes to healthy diets, portion sizes, physical activity and screen time. Regular enquiries regarding diet and physical activity on routine visits will enhance awareness about the need for positive modifications^88^.

## Future directions

A holistic approach to tackle the childhood obesity epidemic needs an array of activities which includes steps like influencing policy makers and legislation, mobilizing communities, restructuring organizational practices, establishing coalitions and networks, empowering providers, imparting community education as well as enriching and reinforcing individual knowledge and skills^90^. Schools, child care facilities and primary health care centers are important settings for implementation of policies and programmes. Relevant attempts may involve specifying the nutrition composition of foods served in school canteens as well as other outlets, supporting requirements for physical education in schools, increasing the availability of physical activity options or the time available to utilize these options, implementing training programs to empower school teachers to provide nutrition or physical education, and providing financial as well as technical support for programmes and services related to weight control^90^. The advantage of setting-based approaches of this type includes the ability to work with a “captive audience” and to also influence social norms within the setting, with possible transfer to behaviour outside of the setting^90^. Of the possible setting-based interventions, there is sufficient evidence to recommend multi component interventions aimed at diet, physical activity, and cognitive change which makes the approach a holistic and efficient one with demonstrable results^91^.

Any attempt to contain the massive epidemic of childhood obesity will only be fruitful if it is supported by sufficient evidence garnered by appropriate research. Though the evidence is growing in this area, significant deficiencies exist in the areas of epidemiologic transitions in childhood obesity, correlations of obesity to cardiovascular risk factors in an Indian setting as well as efficacy of locally designed interventional programmes. Due importance should also be given to identification and assessment of population determinants of childhood obesity. Research in this field should be directed towards enabling early application of such evidence generated to bring in public health policy changes without delay.

## Conclusion

Obesity in adolescents and children has risen to significant levels globally with serious public health consequences. In addition to cardiovascular, emotional and social issues, it poses a serious hazard to the basic health care delivery system. Unless this epidemic is contained at a war footing, the implications of this global phenomenon on future generations will be serious. The reversibility of this disease with suitable intervention strategies should be seen as an opportunity and efforts pursued with vigour.
